# Stability Analysis of the Asiatic Acid-COX-2 Complex Using 100 ns Molecular Dynamic Simulations and Its Selectivity against COX-2 as a Potential Anti-Inflammatory Candidate

**DOI:** 10.3390/molecules28093762

**Published:** 2023-04-27

**Authors:** Ida Musfiroh, Rahmana E. Kartasasmita, Slamet Ibrahim, Muchtaridi Muchtaridi, Syahrul Hidayat, Nur Kusaira Khairul Ikram

**Affiliations:** 1 Department of Pharmaceutical Analysis and Medicinal Chemistry, Faculty of Pharmacy, Universitas Padjadjaran, Bandung 45363, Indonesia; 2 Department of Pharmacochemistry, School of Pharmacy, Institute Technology Bandung, Bandung 40132, Indonesia; 3 Faculty of Pharmacy, Universitas Jenderal Ahmad Yani, Bandung 40285, Indonesia; 4 Institute of Biological Sciences, Faculty of Science, Universiti Malaya, Kuala Lumpur 50603, Malaysia

**Keywords:** asiatic acid, COX-2, IC_50_, molecular dynamic simulation, anti-inflammatory

## Abstract

Asiatic acid, a triterpenoid compound, has been shown to have anti-inflammatory activity through the inhibition of the formation of cyclooxygenase-2 (COX-2) in vitro and in vivo. This study was conducted to determine the binding stability and the inhibitory potential of asiatic acid as an anti-inflammatory candidate. The study involved in vitro testing utilizing a colorimetric kit as well as in silico testing for the pharmacophore modeling and molecular dynamic (MD) simulation of asiatic acid against COX-2 (PDB ID: 3NT1). The MD simulations showed a stable binding of asiatic acid to COX-2 and an RMSD range of 1–1.5 Å with fluctuations at the residues of Phe41, Leu42, Ile45, Arg44, Asp367, Val550, Glu366, His246, and Gly227. The total binding energy of the asiatic acid–COX-2 complex is −7.371 kcal/mol. The anti-inflammatory activity of the asiatic acid inhibition of COX-2 was detected at IC_50_ values of 120.17 µM. Based on pharmacophore modeling, we discovered that carboxylate and hydroxyl are the two main functional groups that act as hydrogen bond donors and acceptors interacting with the COX-2 enzyme. From the results, it is evident that asiatic acid is a potential anti-inflammatory candidate with high inhibitory activity in relation to the COX-2 enzyme.

## 1. Introduction

Inflammation is the response of the immune system to noxious stimuli such as pathogens, damaged cells, toxic compounds, or irradiation [1]. Inflammation is a component of the nonspecific immune response that happens in response to any type of bodily injury. The immune system plays a role in eliminating destructive stimuli and initiating the healing process [2]. The primary symptoms of inflammation are increased blood flow, elevated cellular metabolism, vasodilatation, the release of soluble mediators, the extravasation of fluids, and cellular influx [2]. Inflammation is also characterized by redness of the skin, burning sensation, swelling, loss of function, and pain [3]. COX-2 is an enzyme that plays important roles in response to injury, infection, inflammation, and carcinogenesis [4].

Cyclooxygenase-2 (COX-2), also known as prostaglandin-endoperoxide synthase, is an enzyme that is commonly found in the inflammation site and is responsible for the conversion of arachidonic acid into prostaglandins (PG) and thromboxanes, which are inflammatory mediators [5]. The production of PG from COX-2 is regulated by the transcription factor NF-κB. The overexpression of COX-2 is the common cause of the pathogenesis of inflammatory disease [6].

Non-steroidal anti-inflammatory drugs (NSAIDs) are currently used to treat mild to moderate pain and inflammation through the inhibition of COX-2. However, these medications frequently become ineffective after prolonged use and have undesirable side effects. As a result, drug discovery and development should be performed to develop a drug that is more effective with fewer side effects. Asiatic acid is an active compound from *Centella asiatica* that has been developed as a potentially promising drug candidate due to its various pharmacological properties and fewer side activities [7].

**Figure 1:**
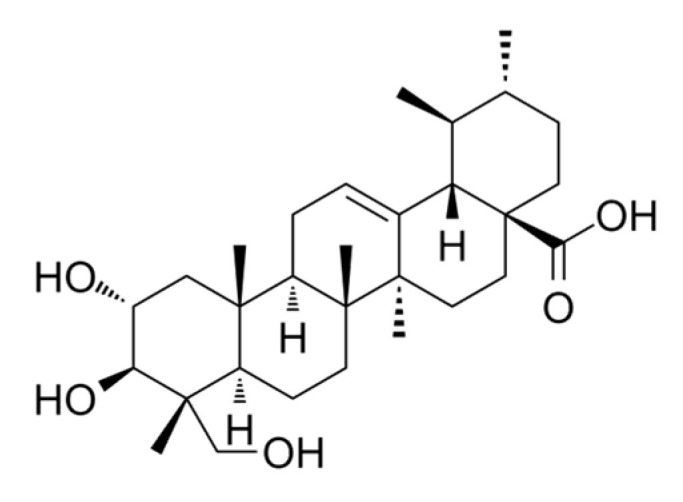
Asiatic acid structure.

Asiatic acid is a pentacyclic triterpenoid compound with high biological activity, such as anti-cancer, hepatoprotector, wound-healing and anti-inflammation properties [8,9,10]. It is also known as 2,3,23-trihydroxy-urs-12-ene-28-oic-acid, an asiaticoside aglycone that can be produced by acidicly hydrolyzing the sugar moiety of the asiaticoside molecule. Its molecular weight and chemical formula are 488.70 kD and C_30_H_48_O_5_, respectively. Asiatic acid is a naturally pentacyclic triterpenoid with three hydroxyl groups at atoms C(2), C(3), and C(23), while the olefin and carboxylic acid groups are at atoms C(12) and C(28), respectively. The molecular structure of asiatic acid is given in Figure 1.

Asiatic acid has been reported to modulate various enzymes, receptors, growth factors, transcription factors, apoptotic proteins, as well as cell signaling cascades linked to pharmacological effects [9,11]. In addition, asiatic acid exhibits anti-inflammatory activity through the product inhibition of inducible nitric oxide synthase (iNOS), cyclooxygenase-2 (COX-2), IL-2, IL-6, IL-1β, as well as TNF-α expression via the decreased activation of NF-κB both in vitro and in vivo [12,13]. Other pentacyclic triterpene compounds, such as oleanolic acid and ursolic acid, have also exhibited anti-inflammatory potential through the inhibition of iNOS and COX-2 [14]. Based on those abilities, asiatic acid is predicted to have a similar activity as oleanolic acid and ursolic acid. Asiatic acid from *Centella asiatica* has been used for a long time in the management of skin scars and chronic ulcers in the pharmaceutical industry [7]. Currently, it is used as an anti-inflammatory agent due to various mechanisms. Inflammatory cells activate the expression of cyclooxygenase-2 (COX-2).

In previous studies, an in silico study using a molecular docking simulation has shown the potential of asiatic acid to bind to the COX-2 enzyme with a free energy binding of -1.73 kcal/mol and hydrogen bond interactions in the COX-2 binding pocket at residues Arg 120 and Tyr 385 [12]. Therefore, further research to determine the binding stability of asiatic acid to COX-2 via molecular dynamic simulation and studying the functional groups that correspond to bind with the COX-2 enzyme through pharmacophore modeling are needed to support these findings. An in vitro assay was also carried out to investigate the anti-inflammatory activity of asiatic acid against COX-2.

## 2. Results

### 2.1. Molecular Dynamic Simulation of Asiatic Acid

**Figure 2:**
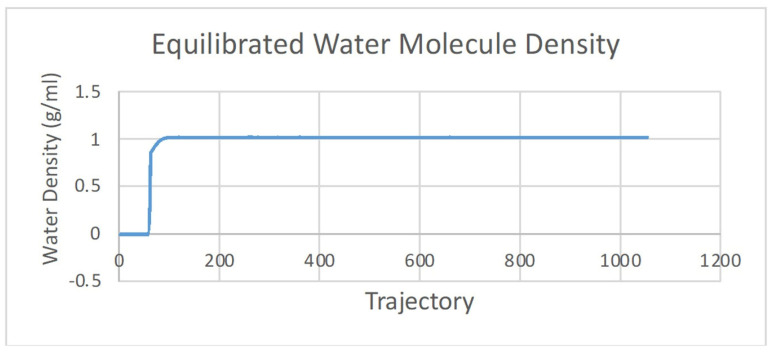
The equilibrated temperature of asiatic acid–COX-2 system.

**Figure 3:**
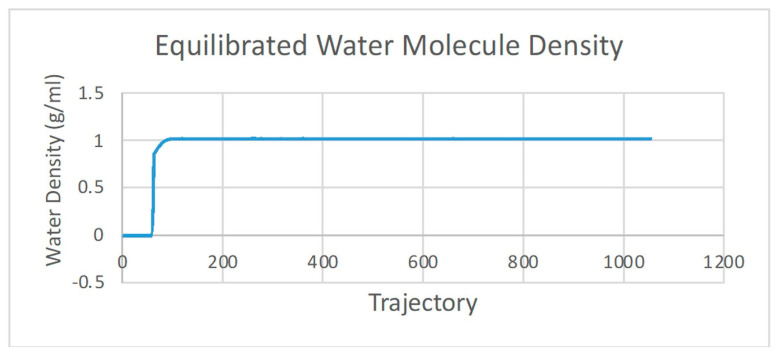
The equilibrated asiatic acid–COX-2 water molecule density.

**Figure 4:**
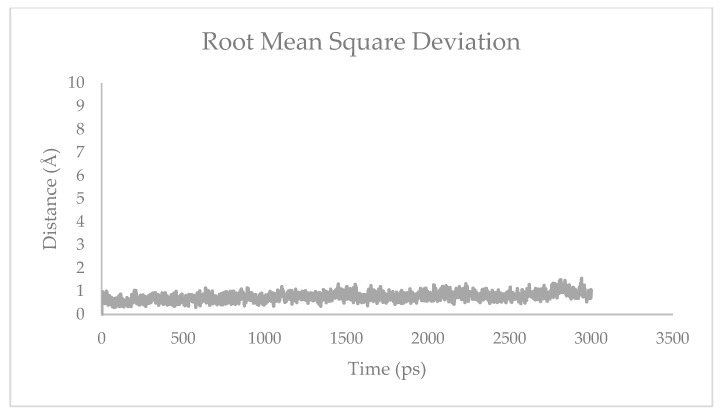
RMSD of asiatic acid–COX-2 system.

**Figure 5:**
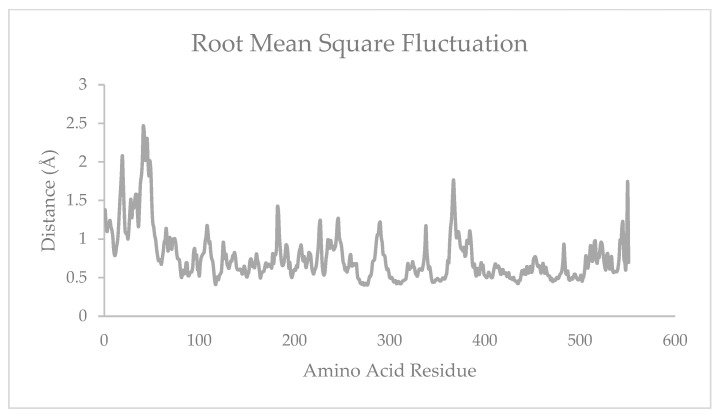
RMSF of asiatic acid–COX-2 system.

Molecular dynamic (MD) simulation is divided into three stages: preparation, production, and analysis [15]. Preparation is an equalizing step of the ligand–receptor complex, which is achieved by adjusting to the conditions of the human body. The parameters include the volume, density, temperature, and pressure. These parameters were equilibrated exactly to the physiology of the human body at 310 K with a water molecular density of 1 g/mL (Figure 2 and Figure 3). The production was performed to predict the stability of the ligand binding to the protein and the potential interactions. An analysis of the stability of asiatic acid–COX-2 complex was carried out by observing the root-mean-square deviation (RMSD) and the root-mean-square fluctuation (RMSF) parameters (Figure 4 and Figure 5). RMSD is a typical measure of the structural distance between coordinates. It calculates the separation between a collection of atoms (e.g., backbone atoms of a protein). The main application of RMSD is to compare the differences between the simulation period’s structures and their reference structure. RMSF is an averaged-over-the-number-of-atoms measurement of an atom or group of atoms’ displacement from the reference structure. The fluctuations of each subset of the structure (each atom, for example) relative to the average structure of the simulation were calculated as RMSF when the simulation is equilibrated [16].

The dynamic interaction strength of asiatic acid with COX-2 was also calculated by evaluating the total binding energy parameters, as illustrated in Table 1.

### 2.2. Pharmacophore Modelling

#### 2.2.1. Pharmacophore Fit Value

The best pharmacophore model (model 7) was made up of substances that have been demonstrated to exhibit activity targeting the COX 2 enzyme. This model was used to compare the pharmacophore similarity of asiatic acid. The pharmacophore fit value was used to evaluate the activity. A better match to the model is indicated by a higher fit score. From this study, asiatic acid has a pharmacophore fit value close to the acetosal value (control) (Table 2). The result is comparable to quercetin, which has a pharmacophore fit score of 36.77 and a docking score of −8.60 kcal/mol, indicating its potential to inhibit the COX-2 enzyme [17]. The docking score of the asiatic acid–COX-2 complex was also comparable to mefenamic acid, with a docking score of −8.90 kcal/mol, and celecoxib, with a docking score of −10.00 kcal/mol and an IC_50_ of 21.58 μg/mL [18]. Based on the results, asiatic acid could be a potential COX-2 inhibitor.

#### 2.2.2. Pharmacophore Modelling and Molecular Interaction

**Figure 6:**
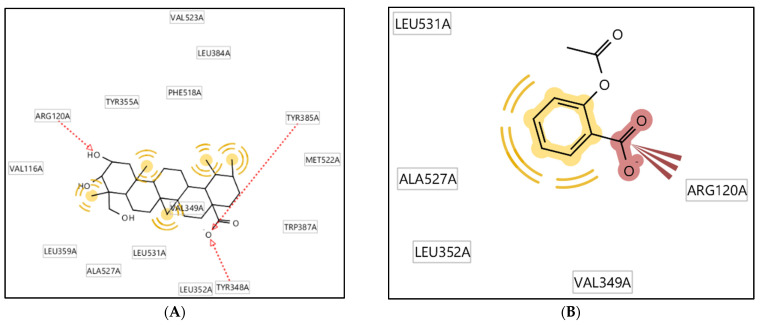
(**A**): Two-dimensional-pharmacophore model of asiatic acid with the Cyclooxygenase Enzyme 2 (COX 2); (**B**): 2D-Pharmacophore model of Acetosal as a lead compound with the Cyclooxygenase Enzyme 2(COX 2).

The arrangement of functional groups that serve as a structure’s active sites was investigated and evaluated in terms of how they interact with the enzymes in molecular interaction studies. The capacity of a molecule to interact with receptors is significantly influenced by the angle and proximity to the conformation of the functional groups that make up the molecule. From the results, we discovered that asiatic acid and acetosal both have a comparable carboxylic acid functional group that acts as a hydrogen bonding acceptor, (Figure 6).

### 2.3. In Vitro Anti-Inflammatory Activity of Asiatic Acid against COX-2

An in vitro assay of COX-2 inhibition using asiatic acid was performed using the Colorimetric COX Inhibitor Screening Assay kit 701050. The principle of measuring the inhibitory activity of the COX enzyme is based on the conversion of the oxidized N,N,N′,N′-tetramethyl-p-phenylenediamine (TMPD) chromogen during the reduction of PGG2 to PGH2. The parameter measured is the oxidized absorbance of TMPD at 590 nm. Due to the heme peroxidase activity, TMPD loses one electron, forming a colored compound that absorbs at 590 nm. The inhibitory activity was measured based on the low absorbance value of the oxidized compound TMPD [19].

The TMPD oxidation is equivalent to the reduction of PGG2 to PGH2 through COX enzyme activity. The higher the COX enzyme activity on the arachidonic acid substrate, the more TMPD is oxidized; hence, the higher the absorbance. In an enzyme-catalyzed reaction, an arachidonic acid substrate will occupy the active site of the COX enzyme, forming a transient enzyme–substrate complex. This complex will be released to produce free COX enzyme and prostaglandin products. The reaction for the formation of this enzyme–substrate complex is reversible. When the reaction takes place, with increasing time, the enzyme will be completely occupied by the arachidonic acid substrate so that the prostaglandin product formed will be relatively constant over a certain period [19].

**Figure 7:**
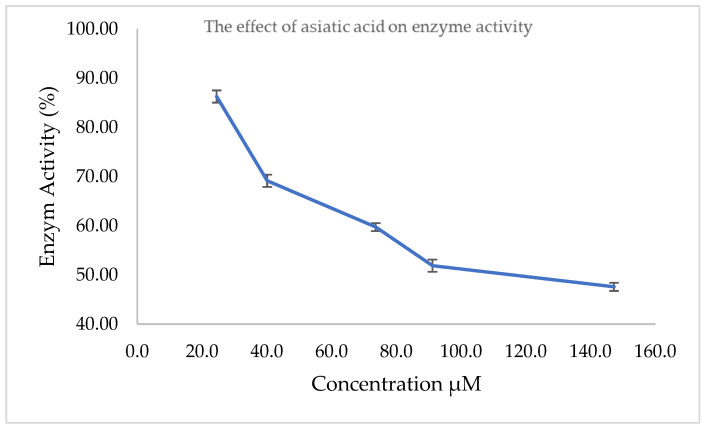
The effect of asiatic acid on enzyme activity.

This assay was performed to support the in silico results of molecular docking and molecular dynamic simulations. Figure 7 shows that asiatic acid has anti-inflammatory activity through COX-2 inhibition with an IC_50_ value of 120.17 µM. The IC_50_ value was obtained from the regression of the % activity curve for the concentration of asiatic acid (Table 3).

## 3. Discussion

### 3.1. Molecular Dynamic Simulation of Asiatic Acid

The stability analysis of the asiatic acid–COX-2 complex was determined through MD simulation. The results illustrated that asiatic acid remains inbound to COX-2, indicating good ligand binding and stability.

The root-mean-square deviation (RMSD) parameter was observed to predict the reproducibility of the MD system performed. The asiatic acid–COX-2 complex presented a low RMSD (Figure 3). This demonstrates that asiatic acid is suitable for the COX-2 binding site, creating good reproducibility. The stability of the asiatic acid–COX-2 complex was achieved after 500 ps with RMSD values of 1–1.5 Å, indicating the high flexibility of asiatic acid–COX-2 interaction.

In the MD simulation, when the ligand binds to the receptor, fluctuations in the amino acids occur due to the dynamic interactions. These fluctuations are represented by the RMSF value, which indicates the flexibility of the amino acid residues. The flexibility of the amino acid residues will facilitate the ligand–receptor interaction because it is not attached to only one position within a certain distance. Therefore, the stability of the bond between the ligand and receptor becomes stronger. The existence of fluctuations in the amino acid residues allows the ligand to bind to the active site of the COX-2 enzyme [20]. The COX-2 active site is divided into three important regions; the first hydrophobic pockets are Tyr385, Trp387, Phe518, Ala201, Tyr248, and Leu352; the second region is the entrance of the active site coated by hydrophilic residues Arg120, Glu524, Tyr355; and the third is a side pocket coated by His90, Arg513, and Val523 [21]. Figure 5 shows the effect of asiatic acid on the flexibility of the amino acid residues on COX-2. In general, asiatic acid results in high residual fluctuations in COX-2. The COX-2 residues that presented the highest fluctuations due to asiatic acid were Phe41, Leu42, Ile45, Arg44, Asp367, Val550, Glu366, His246, and Gly227. This phenomenon indicates that asiatic acid has a low affinity on the active site part of the COX-2 enzyme but is more stable at other parts of the COX-2 enzyme. Based on the energy binding value and the results of the in vitro study, asiatic acid could be an alternative candidate for COX-2 inhibitors via allosteric interaction, which can influence protein or enzyme folding, thus affecting its function [22,23,24].

In addition, the interaction strength of the ligand and receptor was dynamically determined from the total binding energy calculated using the Molecular Mechanism–Poisson–Boltzmann Surface Area (MMPBSA) method. This method calculates the sum of energy from van der Waals interactions, electrostatic energy, electrostatic contribution to solvation free energy, and nonpolar contribution to solvation free energy [25]. Asiatic acid has a total binding energy of -7.371 kcal/mol at the COX-2 receptor.

### 3.2. Pharmacophore Modelling

Compounds that fit the pharmacophore model should also have COX-2 activity as a result of the pharmacophore fit value. Two features may have been disqualified during the virtual screening procedure since not all of the model’s features could be matched. The pharmacophore fit scores would be lower if the features could not be matched. It is interesting to note that asiatic acid’s pharmacophore fit scores (31.25) were close to the lead molecule, acetosal (34.61). This indicates that their chemical properties best matched the pharmacophore model’s characteristics [26]. A greater fit score denotes a better geometric alignment of the chemical properties with the 3D-pharmacophore model. Interestingly, asiatic acid has a lower binding energy (−9.80 kcal/mol) compared to acetosal (−7.00 kcal/mol). The binding energy describes how spontaneously an interaction can occur. Previous studies have shown that quercetin has the potential to inhibit COX-2 enzyme, with a pharmacophore fit score of 36.77 as well as a docking score of −8.60 kcal/mol [17].

The suitability of the pharmacophore features on the ligands makes them easy to interact with, which is associated with lower binding energy values (Table 2). The activation energy decreases with decreasing binding energy, suggesting that little energy is required to establish the contact system between the ligand and the receptor, leading to a spontaneous reaction.

Additionally, the amino acids from the COX-2 enzyme (Arg120, Tyr385, and Tyr348) form the hydrogen bond interactions with the carboxylate and hydroxyl groups of asiatic acid (Figure 6). Moreover, Tyr355, Phe518, Leu384, Val523, Met522, Trp387, Leu352, Ala527, Leu359, and Val116 are amino acid residues that interact with the asiatic acid at the hydrophobic portion. Some of the amino acid residues had a similar pattern to the amino acids in the active site of the COX-2 enzyme. This finding suggests that asiatic acid works similarly to the hydrogen bonding interactions in acetosal compounds, as well as with celecoxib as COX-2-selective inhibitors and non-selective NSAIDs (meloxicam and diclofenac) [21,27].

The hydroxyl, carboxylic, and hydrophobic groups serve as the acceptors or donors of hydrogen bond interaction and hydrophobic interaction with COX-2 in the same active site. The similar interaction between asiatic acid and acetosal suggests that they are positioned at the same active site. As observed in the two structures, the carboxylate group is a pharmacophore structure essential for interaction with the COX-2 enzyme. Based on this pharmacophore modeling, the methyl groups and benzenes of the asiatic acid structure are the parts that can be modified to increase its activity and physiochemical properties. These results are in line with a previous QSAR study that indicated that the hydrophobic interactions of COX-2 inhibitors are important in Cyclooxygenase-2 (COX-2) enzyme inhibition activity [28].

### 3.3. In Vitro Anti-Inflammatory Activity of Asiatic Acid against COX-2

Based on the results, asiatic acid exhibited anti-inflammatory activity by inhibiting the COX-2 activity with an IC_50_ of 120.17 µM. The IC_50_ value was obtained from the regression of the % activity curve for the concentration of asiatic acid. The result is in line with previous studies that reported the anti-inflammatory activity of asiatic acid through the inhibition of edema formation in rats induced by carrageenan at doses of 1, 5, and 10 mg/kg BW intraperitoneally [29]. The study also presented the suppression of proinflammatory mediators, such as COX-2, TNF-α, and IL-1β, which are regulated by the transcription factor, Nf-κB. Asiatic acid also exhibited inhibitory activity against iNOS enzymes, COX-2, and Nf-κB protein expression at 10 mg/kg BW intraperitoneally. Moreover, asiatic acid has better inhibitory activity than indomethacin at 10 mg/kg BW and inhibits several neutrophil cells [29].

Although asiatic acid exhibits the inhibitory activity of the COX-2 enzyme with an IC_50_ value of 120.17 µM, the activity is still considered to be minimal when compared to mefenamic acid, with a docking score of −8.90 kcal/mol, and celecoxib, with a docking score of −10.00 kcal/mol and an IC_50_ of 21.58 μg/mL. Another reported piece of data on the molecular docking of asiatic acid was a Gibbs energy value (∆G⁰) of −1.73 kcal/mol [12]. Based on the data, asiatic acid could be a potential COX-2 inhibitor; however, it might not be selective to the COX-2 enzyme. A previous study shows that coxibs and tNSAIDs, acting as an anti-inflammatory through the inhibition of the COX-2 enzyme, are associated with an increased risk of cardiovascular disease and upper gastrointestinal complications [30]. The inhibitory activity of asiatic acid and COX-2 enzyme occurs through the formation of hydrogen bonds with Arg 120 and Tyr385 at the hydrophilic and hydrophobic pocket of the COX-2 enzyme’s active sites, respectively. The natural ligand of the cyclooxygenase enzyme, arachidonic acid, interacts with COX-2 through a hydrogen bond interaction between the carboxylic group of arachidonic acid, Ser530, and Tyr385 on the COX-2 active site. From this, it is postulated that the anti-inflammatory mechanism of asiatic acid does not occur due to selective COX-2 enzyme inhibition [12] but through the inhibition of Nf-kB, which is a transcription factor for COX-2 enzyme regulation [29].

## 4. Materials and Methods

### 4.1. Materials

The materials used in this research are asiatic acid (97%) from *Centella asiatica* (Sigma Aldrich, St. Louis, MO, USA), arachidonic acid (Cayman Chemical, Ann Arbor, MI, USA), COX-2 enzyme-ovine (Cayman Chemical, Ann Arbor, MI, USA), tris-HCl buffer, and a Colorimeter COX Inhibitor Screening Assay Kit 701050 (Cayman Chemical, Ann Arbor, MI, USA). The pieces of software used in this research are the AMBER18 program, ChemDraw 2D Ultra 12.0 program, LiganScout 4.4.3, MM2 by ChemDraw 3D Software, Gaussian03 Software (Gaussian, Wallingford, CT, USA), Accelrys Discovery Studio 2.5 Software (Accelrys Inc., San Diego, CA, USA), Accelrys Discovery Studio Visualizer 3.5 Software (Accelrys Inc.), AMBER 11 Software (Amber, San Francisco, CA, USA), and AmberTools 1.5 Software (Amber). The pieces of hardware used are an is-nodes Linux cluster, each powered by an Intel Xeon 2.4 GHz processor and 2 GB of RAM (Intel, Santa Clara, CA, USA), an incubator (Memert, Schwabach, Germany), and a plate reader.

### 4.2. Molecular Dynamic Simulation of Asiatic Acid

Molecular dynamics simulations were performed using the AMBER18 program. This simulation was carried out on the asiatic acid compound against the COX-2 enzyme (PDB ID: 3NT1) [31]. The three steps in the molecular dynamics simulation are system preparation, production, and analysis. Appropriate systems with controlled parameters and stability need to be established before the simulation. The preparation was performed by minimizing and equilibrating the system with the specific command “pmemd”. The system was also heated to reach a temperature of 310 K. Equilibration was monitored by determining the stability of the temperature, energy, and density of the system as well as root-mean-square deviation. The molecular dynamics simulations were carried out to ensure the stability of the complex in a system that is identical to the human body (temperature, 310 K; pressure, 1 atm; and volume, 1 g/mL). Protein–ligand complex visualization and MD trajectory analysis were performed using VMD software. The complex binding affinity was calculated using the MMGBSA method [15].

### 4.3. Pharmacophore Modelling

#### 4.3.1. Structure Preparation

The 2D structure of asiatic acid was generated using the ChemDraw 2D Ultra 12.0 program, and the energy was minimized using MM2 by ChemDraw 3D software, and then the structure was saved in the pdb format [32,33].

#### 4.3.2. Database Preparation

Pharmacophore modeling was obtained from the Active Compound Database, Decoy Compound Database, and Test Compound Database. The active and decoy compounds were downloaded from http://dude.docking.org, (accessed on 2 August 2022) whereas the test compounds were obtained from the previous preparation procedure. Then, each one was opened with the type of “training” compound for Active and Decoy and the type of “test” compound for the test compound using LigandScout 4.4.3. Following that, the databases are saved in the .ldb format [34].

#### 4.3.3. Creating Pharmacophore

Pharmacophore modeling was carried out using Ligand Scout 4.4.3. The predefined active compound database file was opened, and the ligand-based menu was then sorted by cluster. Each cluster consists of one or more compounds, one of which must be transformed into a training compound for each cluster, with the other compounds being transformed into the type “ignored”. The top ten pharmacophore models were then validated once the pharmacophore model had been generated [34].

#### 4.3.4. Validation Pharmacophore

To determine the best pharmacophore model, ten of the models obtained were evaluated. Each pharmacophore model, as well as the database for active and decoy drugs, were included in the screening column. Then, pharmacophore screening was performed by clicking the “screening perspective” button. To evaluate the efficacy of the pharmacophores, the Receiver Operating Characteristic (ROC) curve was employed [34].

#### 4.3.5. Screening Test Compounds

The screening column was loaded with the previously produced test compound database. The top pharmacophore model from the ligand-based portion was then forwarded to the screening column for additional processing to identify the top molecule based on the highest pharmacophore fit score [34].

### 4.4. In Vitro Anti-Inflammatory Activity of Asiatic Acid against COX-2

A total of 160 μL of tris-HCl buffer and 10 μL of heme were added to 3 wells as background wells. Then, 150 μL of tris-HCl buffer, 10 μL of heme, and 10 μL of the enzyme were included in 3 wells as the 100% initial activity wells. Then, 150 μL of tris-HCl buffer, 10 μL of heme, 10μ L of the enzyme, and 10 μL of the test sample were added into the inhibitor well to reach a final concentration of 68.182 ppm, 45.454 ppm, and 22.727 ppm. Then, 10 μL of solvent was added to the 100% initial activity wells and background wells. The plate was agitated for a few seconds and incubated for 5 min at 25 °C. Then, 20 μL of colorimetric substrate solution was added to all wells. Then, 20 μL of arachidonic acid was then added to the wells. The plate was agitated carefully for a few seconds and incubated for 5 min at 25 °C. The absorbance readings were carried out at 590 nm using a plate reader. The parameter of the plate reader is capable of measuring the absorbance between 590 and 611 nm [19].

## 5. Conclusions

From the in silico and in vitro study, it can be concluded that asiatic acid has potential as an anti-inflammatory agent. The in silico studies through the use of MD simulation showed asiatic acid binding stability to COX-2, with RMSD values of 1–1.5 Å and fluctuations in the residues Phe41, Leu42, Ile45, Arg44, Asp367, Val550, Glu366, His246, and Gly227. The fluctuation represents allosteric interaction (or allosteric control), which is the regulation of an enzyme through binding to the effector molecule at a location other than the active site of the enzyme. The total binding energy value is −7.3710794 kcal/mol, indicating the binding strength of the asiatic acid–COX-2 complex. Based on pharmacophore modeling, we also discovered that the major functional groups of asiatic acid that interact with the COX-2 enzyme were its carboxylate, hydroxyl, and hydrophobic functional groups. The results were also supported by in vitro assays. The inhibitory activity of asiatic acid against COX-2 resulted in an IC50 value of 120.17 µM. The overall finding suggests that asiatic acid could be a promising COX-2 inhibitor with high efficacy.

## Figures and Tables

**Table 1:** The total binding energy of asiatic acid–COX-2.

Components (kcal/mol)	Asiatic Acid–COX-2
Van der Waals Interaction	−3.891
Electrostatic Energy	−39.870
Electrostatic Contribution to Solvation-Free Energy (E_GB_)	−4.928
Nonpolar Contribution to Solvation-Free Energy (E_SURF_)	177.4419
ΔGgas (VdW + E_EL_)	−2.619
ΔGsolv (E_GB_ + E_SURF_)	−4.751
ΔG_TOTAL_ (VdW + E_EL_ + E_GB_ + E_SURF_)	−7.371

**Table 2:** LigandScout pharmacophore fit score of asiatic acid retrieved using the 3D-structure-based pharmacophore derived from acetosal bound to the Cycloxigenase enzyme 2.

Compound	Pharmacophore-Fit Score	Energy (kcal/mol)
Acetosal	34.61	−7.00
Asiatic acid	31.25	−9.80

**Table 3:** Anti-inflammatory activity of asiatic acid against the COX-2 enzyme.

No.	Concentration (μM)	% Enzyme Activity	SD
1	24.5	86.27	0.012
2	40.1	69.15	0.012
3	73.64	59.74	0.008
4	91.18	51.89	0.012
5	147.27	47.58	0.008
IC_50_	120.17		

## Data Availability

The data analysed or generated during the study are included in this article.

## Sample Availability

Samples of the compounds are available from the authors.
